# From vital sign trajectories to data-driven targets: defining exploratory blood gas ranges in sepsis-associated thrombocytopenia

**DOI:** 10.3389/fmed.2026.1801744

**Published:** 2026-04-09

**Authors:** Shihao Jin, Haotian Hu, Xue Wang, Xuan Wei, Changjie Wang, Peisen Ding, Pengfei Fan, Sinan Gao, Xiaojing Dou, Bing Wang

**Affiliations:** 1 Surgical Intensive Care Unit, Tianjin First Central Hospital, Tianjin, China; 2 Department of Science, Technology and Education, Tianjin First Central Hospital, Tianjin, China

**Keywords:** critical care, data-drive, precision medicine, sepsis-associated thrombocytopenia, trajectory phenotyping

## Abstract

**Background:**

The early in-intensive care unit (ICU) phase is critical for sepsis-associated thrombocytopenia (SATP) patients, yet the prognostic value of their initial physiological trajectory remains underexplored. We aimed to identify distinct subgroups based on vital sign trajectories following ICU admission and to investigate their differential outcomes and subsequent blood gas management needs.

**Methods:**

This retrospective study utilized the MIMIC-IV database. Adults with SATP were included. Group-based multi-trajectory modeling (GBMTM) was applied to hourly vital signs (including heart rate, blood pressure, respiratory rate, and SpO₂) from the first 12 h of ICU stay to identify subgroups. Mortality risk was assessed using Cox regression, with the lowest-risk cluster as the reference. Within the identified high-risk sub-phenotype, the nonlinear relationships between blood gas ranges and ICU mortality were analyzed with restricted cubic splines (RCS). Finally, multivariable partial dependence plots (PDP) were employed to quantify the optimal ranges for blood gas parameters, defined as those associated with the lowest ranges of predicted mortality risk for this subgroup.

**Results:**

The analysis of initial 12-h physiological trajectories classified patients into three subgroups: Cluster 1 (characterized by elevated blood pressure), Cluster 2 (marked by high heart rates and respiratory rates with low SpO₂), and Cluster 3 (low blood pressure with high SpO₂). Cluster 2 was identified as the high-risk subgroup, demonstrating significantly increased mortality risks compared with Cluster 3: ICU mortality (HR = 1.40; 95% CI: 1.13–1.73), 28-day mortality (HR = 1.56; 95% CI: 1.30–1.88), 90-day mortality (HR = 1.43; 95% CI: 1.21–1.67), and 365-day mortality (HR = 1.33; 95% CI: 1.15–1.54). Within Cluster 2, restricted cubic spline analyses revealed nonlinear relationships between blood gas parameters and ICU mortality. Using partial dependence plot analysis, we identified model-derived ranges of blood gas values associated with the lowest predicted mortality risk, which may serve as exploratory physiological references for this high-risk subgroup: pH 7.32–7.64, PO₂ 25.00–324.32 mmHg, PCO₂ 21.94–53.74 mmHg, lactate 0.6–7.49 mmol/L, base excess −7.47 to 23.00 mEq/L, and total CO₂ 43.47–56.00 mEq/L. These ranges, though broad, reflect the inherent physiological variability during the early ICU phase and should be interpreted as hypothesis-generating parameters rather than strict clinical targets.

**Conclusion:**

Early vital sign trajectories during the first 12 h in the ICU effectively stratify SATP patients into prognostic subgroups. For the high-risk subphenotype, we further delineated model-derived physiological ranges of blood gas parameters, creating a “trajectory-to-targets” framework. This approach offers a hypothesis-generating strategy for transitioning from early risk identification to personalized physiological insights in the critical early phase of ICU care.

## Introduction

Sepsis represents a dysregulated host response to infection that culminates in life-threatening organ dysfunction (1–4), and stands as a leading cause of mortality among critically ill patients in the intensive care unit (ICU) (1, 4, 5). Within this pathophysiological landscape, excessive inflammatory reactions drive both heightened platelet consumption and impaired platelet production, frequently resulting in thrombocytopenia (6). Sepsis-associated thrombocytopenia (SATP) manifests in 35–59% of septic patients (7–9) and is closely linked to adverse outcomes, with studies attributing 13–83% of sepsis-related mortality to its presence (6, 10). SATP exacerbates prognosis by increasing risks of hemorrhage, transfusion dependency, need for life-support interventions, prolonged hospitalization, and overall mortality (11, 12).

In the ICU environment, high-resolution temporal monitoring of vital signs affords a dynamic, multidimensional perspective on patient physiology, proving instrumental in discerning subpopulations with divergent pathophysiological mechanisms and clinical trajectories (13, 14). Indeed, trajectories derived from bedside vital signs have previously enabled the identification of sepsis subgroups with distinct outcomes and therapeutic responses (15). Although prior studies have utilized unsupervised methods on electronic health record data and clinical trial biomarkers to define between two and six subgroups, these efforts have largely relied on static clinical value, which may fail to capture phenotypes consistent over time (16). In contrast, longitudinal data offer a more robust foundation for identifying SATP subgroups that differ meaningfully in clinical evolution, outcome, and treatment responsiveness (17, 18). Notably, septic patients with concomitant thrombocytopenia experience significantly worse prognoses than those without (19). Nevertheless, the relationship between early vital sign dynamics and clinical outcomes within this high-risk SATP population remains systematically uncharacterized.

This study was designed to identify early, clinically relevant SATP subgroups using dynamic vital sign trajectories captured within the first 12 h following ICU admission. Our study was structured around three key objectives. First, to establish trajectory-based SATP subgroups firmly rooted in early vital signs data; second, to characterize these subgroups according to clinical presentation, illness severity, and prognostic profile; and third, to investigate the role of blood gas parameters, with a specific focus on the association between their value ranges and ICU mortality within the identified high-risk subgroup.

## Methods

### Data source

Data were sourced from the Medical Information Mart for Intensive Care IV (MIMIC-IV, v 3.1) database (20), a publicly available, de-identified repository comprising comprehensive health records of patients admitted to ICUs at the Beth Israel Deaconess Medical Center between 2008 and 2022. MIMIC-IV includes detailed demographic, vital sign, laboratory, comorbidity, treatment, and outcome data, enabling robust analyses of critically ill populations. One author (SJ) completed the National Institutes of Health web-based course “Protecting Human Research Participants” (Record ID: 66200187) and obtained approval to extract data. All patient identifiers were removed, and the institutional ethical committee waived the requirement for informed consent. This study adheres to the principles of the Declaration of Helsinki. This retrospective cohort study is reported in accordance with the Strengthening the Reporting of Observational Studies in Epidemiology (STROBE) guidelines. The completed STROBE checklist is provided in Supplementary File S1.

### Study population

We included adult patients (aged 18–89 years) diagnosed with SATP during their first ICU admission. Sepsis was identified using ICD-9 and ICD-10 codes (99592, A419, R6521, R6520, A4159, and A4150), and thrombocytopenia was identified using ICD codes (2875, D696, D6959, 28749, 2874, and 2841) (21). Details of the inclusion/exclusion process are provided in Supplementary Figure S1. For those with multiple ICU admissions, only data from the first admission were analyzed. Survival status was determined using hospital records and linkage to the Social Security Death Index, which provides comprehensive mortality follow-up for the majority of patients.

### Data extraction

Data from the first 24 h post-admission were extracted, including: (1) demographics, including gender and age; (2) comorbidities, including hypertension, type 2 diabetes mellitus, heart failure, chronic kidney disease and pneumonia; (3) vital signs, including heart rate (HR), respiratory rate (RR), systolic blood pressure (SBP), diastolic blood pressure (DBP), mean arterial pressure (MAP), and oxygen saturation (SpO_2_); (4) baseline laboratory tests, including complete blood count: white blood cell count (WBC), red blood cell count (RBC), platelet count, hemoglobin and red cell distribution width (RDW); comprehensive metabolic panel: total bilirubin, anion gap, chloride, potassium, sodium, calcium, glucose, creatinine and blood urea nitrogen (BUN); blood gas: pH, lactate, arterial partial oxygen pressure (PO_2_), base excess, total pressure of carbon dioxide (total PCO_2_) and partial pressure of carbon dioxide (PCO_2_); coagulation function: prothrombin time (PT), partial thromboplastin time (PTT) and international normalized ratio (INR) and (5) treatments and scores, including mechanical ventilation, renal replacement therapy, as well as sequential organ failure assessment (SOFA) score were collected on ICU day one. To maximize statistical power, all eligible patients from MIMIC-IV were included. Variables with >30% missing values were excluded.

### Group-based multi-trajectory model development

Vital signs (HR, RR, SBP, DBP, MAP, and SpO₂) were aggregated hourly over the first 12 h post-ICU admission, with multiple measurements averaged per hour. All values were standardized using *Z*-scores to minimize scale-related bias. Group-based multi-trajectory modeling (GBMTM) was applied to identify SATP subgroups based on multivariate vital sign trajectories. This unsupervised algorithm detects distinct trajectory patterns using finite mixture models with polynomial regression, with parameters estimated via expectation–maximization (22). Unlike single-variable trajectory models, GBMTM clusters patients using multiple longitudinal markers, offering a more holistic view of disease progression (23, 24). Standardized data were used to derive SATP subgroups characterized by unique polynomial functions describing temporal vital sign trends.

To determine the optimal number of trajectory groups, we fitted models specifying one to six groups and evaluated them based on a combination of statistical fit indices and clinical interpretability (Supplementary Table S2). The selection criteria included: (i) the Bayesian information criterion (BIC) and Akaike information criterion (AIC), where lower absolute values indicate better model fit; (ii) Entropy, a measure of classification accuracy, with values > 0.7 suggesting clear separation between groups; and (iii) group size, requiring each identified cluster to contain at least 5% of the total sample to ensure clinical relevance and sufficient statistical power for subsequent analyses. Based on these criteria, the three-group solution was selected as it exhibited a substantial drop in BIC relative to the two-group model, achieved an entropy of 0.96, and maintained adequate group sizes (Cluster 1: 23.99%, Cluster 2: 35.36%, Cluster 3: 40.65%).

For the selected three-group model, the polynomial order (linear, quadratic, or cubic) for each vital sign trajectory was determined by backward elimination. We initially specified a cubic function for all trajectories and then removed higher-order terms that were not statistically significant, thereby retaining the most parsimonious specification that adequately captured the temporal trend. Model adequacy was further confirmed by calculating the average posterior probability (AvePP) for assignment to each group; all groups had AvePP >0.7, indicating high classification certainty. Additionally, the odds of correct classification (OCC) exceeded 5.0 for all groups, confirming the model’s discriminant validity.

### Statistical analysis

Adjusted Kaplan–Meier curves, stratified by age, sex, and race, were used to compare 365-day mortality. Hazard ratios (HRs) were estimated via Cox proportional hazards models, adjusted for additional covariates. For the phenotype with the highest mortality, a *post-hoc* analysis was conducted to explore potential physiological dysregulation. Restricted cubic splines (RCS) modeled dose–response relationships between blood gas ranges and ICU mortality. Random forest (RF) classifiers were built to predict ICU mortality within high-risk subgroups, using clinical characteristics and blood gas values. Hyperparameters were tuned via fivefold cross-validation and multi-predictor interactive partial dependence plots (PDPs) visualized relationships between blood gas ranges and mortality risk. For the subsequent partial dependence plot (PDP) analysis, we adopted a univariate approach in which each blood gas parameter was modeled independently.

To ensure data quality, we applied a multi-step preprocessing protocol. First, physiologically implausible values were identified using clinically defined range checks and set to missing. Second, to mitigate the influence of extreme but clinically plausible outliers, we winsorized all continuous variables at the 1st and 99th percentiles. Third, missing data were handled using linear interpolation for gaps ≤2 h and last-observation-carried-forward for isolated missing points; patients with >4 consecutive hours of missing data were excluded from trajectory modeling.

Data extraction was performed using SQL in PostgreSQL (v 15). All analyses were conducted in R (v 4.3.1). Normally distributed variables are presented as mean ± standard deviation (SD), while non-normally distributed variables are presented as median with interquartile range (IQR). Categorical variables are presented as counts and percentages. A two-tailed *p*-value <0.05 was considered statistically significant.

## Results

### Baseline characteristics

**Figure 1:**
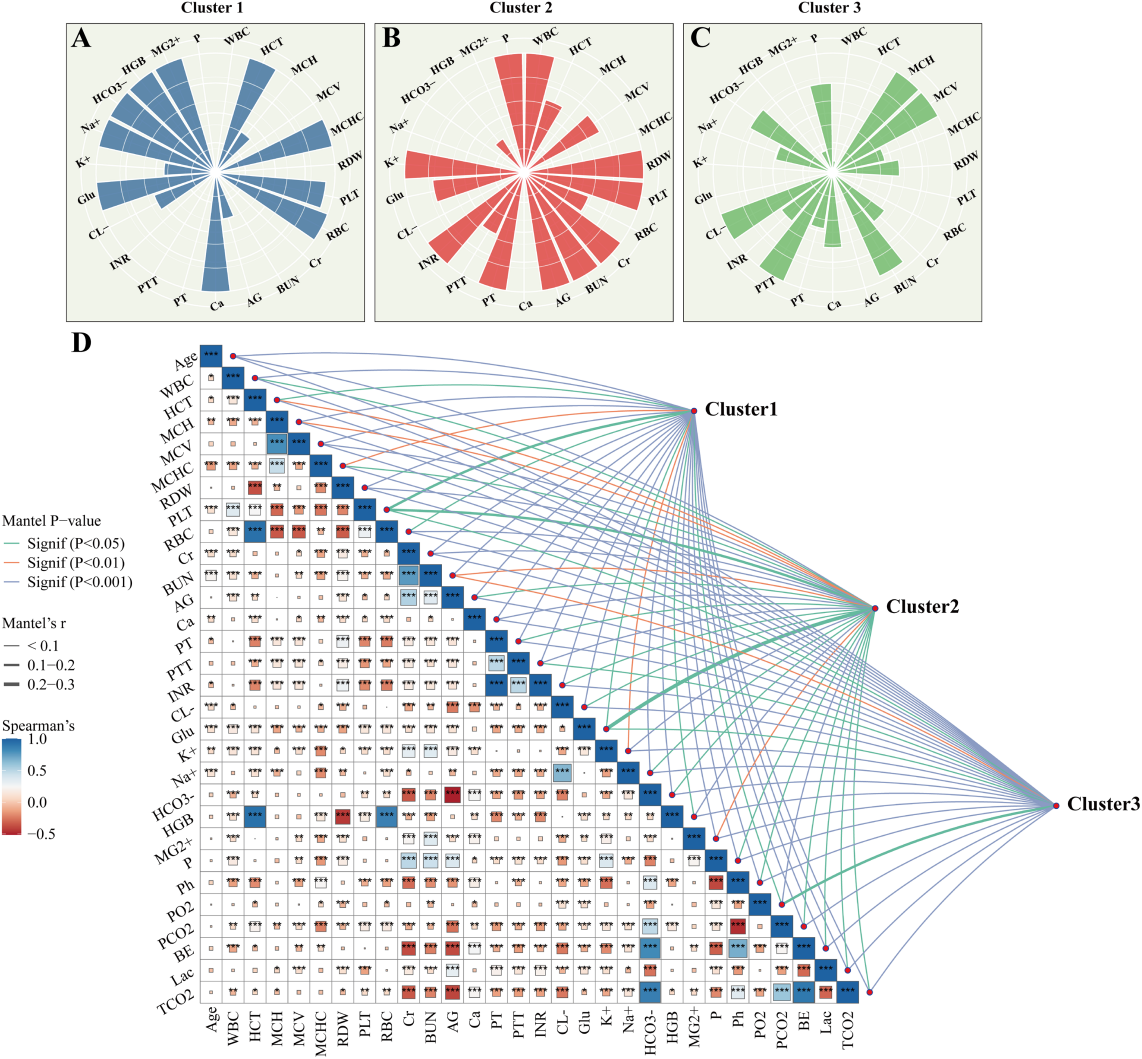
Clinical characteristics among different subgroups. **(A–C)** Radar charts of relative levels of hematological, biochemical, and electrolyte variables within the three clusters. Values have been standardized using *Z*-scores for clarity. **(D)** Spearman correlation matrix between clinical variables and subgroups. In the plot, the triangular section displays the correlations between variables. Darker (blue) squares indicate positive correlations, while lighter (orange) squares indicate negative correlations. The size of each square corresponds to the absolute value of the correlation coefficient. The upper-right section depicts the correlation between phenotypes and clinical characteristics. The color indicates the significance of the correlation, while the width represents the correlation coefficients. ^*^p < 0.05, ^**^p < 0.01, and ^***^p < 0.001.

From an initial cohort of 12,126 adults with SATP, we excluded 9,929 patients due to missing or anomalous data, resulting in 2,197 eligible subjects for final analysis (Supplementary Figure S1). Clinical and demographic characteristics across the three identified subgroups are summarized in Table 1, Figure 1, and Supplementary Table S1. Cluster 1 was characterized by the highest baseline blood pressure, designating it the hypertensive subgroup. Cluster 2 was characterized by elevated heart rate, tachypnea, reduced SpO₂, and upregulated inflammatory markers (WBC, RDW, anion gap, lactate, and BUN), thereby classifying it as the hyperinflammatory subgroup. In contrast, Cluster 3 demonstrated the lowest values for heart rate, respiratory rate, mean blood pressure, WBC, lactate, PT, and INR, supporting its classification as a hypo-inflammatory subgroup (Table 1; Figure 1; and Supplementary Table S1). Temporal dynamics further confirmed distinct vital sign distributions among subgroups (Supplementary Figure S5).

**Table 1:** The clinical characteristics of subgroups.

Variables	All	Cluster 1 (*N* = 527)	Cluster 2 (*N* = 777)	Cluster 3 (*N* = 893)	*p*-value
Age (year)	63.94 ± 14.93	61.63 ± 14.43	61.55 ± 15.42	67.39 ± 14.12	**<0.001**
Male	1,312 (59.72%)	314 (59.60%)	451 (58.00%)	547 (61.30%)	0.41
Hospital length of stay (day)	19.04 ± 17.29	20.59 ± 18.05	19.57 ± 18.82	17.66 ± 15.24	**0.005**
ICU length of stay (day)	8.66 ± 9.67	9.01 ± 10.22	9.63 ± 10.53	7.60 ± 8.37	**<0.001**
Hospital mortality	658 (29.95%)	122 (23.10%)	319 (41.10%)	217 (24.30%)	**<0.001**
ICU mortality	490 (22.30%)	87 (16.50%)	251 (32.30%)	152 (17.00%)	**<0.001**
28-day mortality	632 (28.77%)	119 (22.60%)	297 (38.20%)	216 (24.20%)	**<0.001**
90-day mortality	848 (38.6%)	167 (31.70%)	371 (47.70%)	310 (34.70%)	**<0.001**
365-day mortality	1,016 (46.24%)	204 (38.70%)	422 (54.30%)	390 (43.70%)	**<0.001**
Smoker	179 (8.15%)	46 (8.70%)	68 (8.80%)	65 (7.30%)	0.47
Alcohol abuse	33 (1.5%)	8 (1.50%)	16 (2.10%)	9 (1.00%)	0.21
Hypertension	805 (36.64%)	205 (38.90%)	278 (35.80%)	322 (36.10%)	0.46
Diabetes	700 (31.86%)	165 (31.30%)	232 (29.90%)	303 (33.90%)	0.20
Myocardial infarction	367 (16.7%)	76 (14.40%)	117 (15.10%)	174 (19.50%)	**0.02**
Congestive heart failure	647 (29.45%)	127 (24.10%)	214 (27.50%)	306 (34.30%)	**<0.001**
Mild liver disease	622 (28.31%)	139 (26.40%)	232 (29.90%)	251 (28.10%)	0.39
Severe liver disease	340 (15.48%)	68 (12.90%)	121 (15.60%)	151 (16.90%)	0.13
Renal disease	509 (23.17%)	121 (23.00%)	140 (18.00%)	248 (27.80%)	**<0.001**
Chronic pulmonary disease	602 (27.4%)	139 (26.40%)	213 (27.40%)	250 (28.00%)	0.80
Cerebrovascular disease	253 (11.52%)	75 (14.20%)	82 (10.60%)	96 (10.80%)	0.08
Peripheral vascular disease	218 (9.92%)	29 (5.50%)	73 (9.40%)	116 (13.00%)	**<0.001**
Dementia	81 (3.69%)	14 (2.70%)	24 (3.10%)	43 (4.80%)	0.06
Peptic ulcer disease	100 (4.55%)	25 (4.70%)	33 (4.20%)	42 (4.70%)	0.88
Paraplegia	71 (3.23%)	30 (5.70%)	14 (1.80%)	27 (3.00%)	**<0.001**
Malignant cancer	396 (18.02%)	104 (19.70%)	163 (21.00%)	129 (14.40%)	**0.001**
Metastatic solid tumor	180 (8.19%)	33 (6.30%)	85 (10.90%)	62 (6.90%)	**0.002**
AIDS	21 (0.96%)	7 (1.30%)	11 (1.40%)	3 (0.30%)	**0.05**
Rheumatic disease	78 (3.55%)	26 (4.90%)	29 (3.70%)	23 (2.60%)	0.06
Invasive ventilation	1,725 (78.52%)	411 (78.00%)	606 (78.00%)	708 (79.30%)	0.77
CRRT	385 (17.52%)	75 (14.20%)	184 (23.70%)	126 (14.10%)	**<0.001**
RRT	469 (21.35%)	104 (19.70%)	209 (26.90%)	156 (17.50%)	**<0.001**

Bold for statistical significance.

### Vital sign trajectories and derivation of SATP subgroups

**Figure 2:**
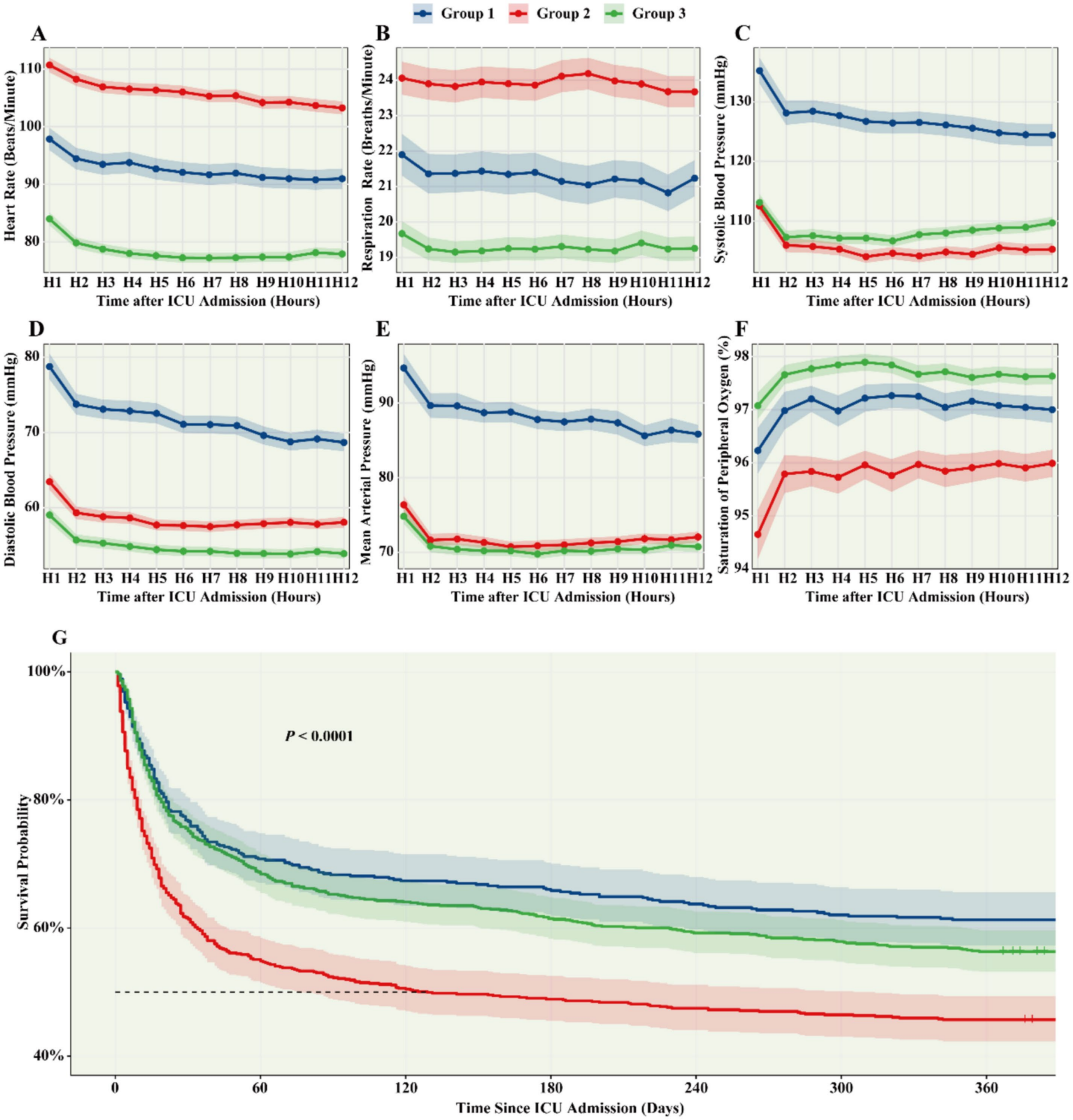
Group-based trajectory modeling of vital signs and adjusted Kaplan–Meier plots. **(A–F)** Group-level trajectories of vital signs over the first 12 h. **(G)** Adjusted Kaplan–Meier curves for 365-day mortality across subgroups, with shading indicating 95% confidence intervals. Curves were adjusted for age, sex, and race.

Vital sign trajectories over the first 12 h post-ICU admission revealed substantial inter-patient heterogeneity, with trajectories frequently intersecting, underscoring the variability in clinical progression among SATP patients (Supplementary Figure S2). Consensus clustering identified *K* = 3 as the optimal number of subgroups (Supplementary Figures S3, S4), a finding consistently supported by sensitivity analyses across *K* = 2 to 6 (Supplementary Table S2). Thus, a three-class GBMTM was selected as the final model (Figures 2A–F).

Cluster 2 exhibited the highest ICU mortality rate and the longest median ICU length of stay, while Clusters 1 and 3 showed comparatively lower ICU mortality (Table 1). In the survival analyses using Cluster 3 as the reference group, Cluster 2 was consistently associated with significantly higher mortality risks across all time points. After full adjustment for age, sex, SOFA score, APS-III score, invasive ventilation, CRRT, and RRT (Model 2), Cluster 2 demonstrated a 1.40-fold increased risk of ICU mortality (95% CI: 1.13–1.73; *p* = 0.002), a 1.56-fold increased risk of 28-day mortality (95% CI: 1.30–1.88; *p* < 0.001), a 1.43-fold increased risk of 90-day mortality (95% CI: 1.21–1.67; *p* < 0.001), and a 1.33-fold increased risk of 365-day mortality (95% CI: 1.15–1.54; *p* < 0.001) compared with Cluster 3. Similar patterns were observed in the crude models and in models adjusted only for age and sex (Model 1), with all hazard ratios for Cluster 2 remaining statistically significant (all *p* < 0.05). These findings indicate that Cluster 2 represents a high-risk subgroup with substantially elevated short- and long-term mortality, whereas Cluster 1 shows comparable prognosis to the reference group (Figure 2G and Table 2).

**Table 2:** Cox regression result between subgroups of ICU/28/90/365-day mortality.

Cluster 3		Cluster 1		Cluster 2	
		HRs (95% CI)	*p*-value	HRs (95% CI)	*p*-value
ICU mortality					
Crude	Ref.	0.82 (0.63, 1.07)	0.143	**1.50 (1.23, 1.84)**	**<0.001** ^a^
Model 1^b^	Ref.	0.91 (0.70, 1.18)	0.477	**1.76 (1.43, 2.16)**	**<0.001**
Model 2^c^	Ref.	0.94 (0.72, 1.23)	0.668	**1.40 (1.13, 1.73)**	**0.002**
28-day mortality					
Crude	Ref.	0.93 (0.74, 1.16)	0.518	**1.79 (1.50, 2.13)**	**<0.001**
Model 1	Ref.	1.09 (0.87, 1.37)	0.432	**2.12 (1.78, 2.54)**	**<0.001**
Model 2	Ref.	1.16 (0.93, 1.45)	0.199	**1.56 (1.30, 1.88)**	**<0.001**
90-day mortality					
Crude	Ref.	0.90 (0.75, 1.09)	0.291	**1.59 (1.37, 1.85)**	**<0.001**
Model 1	Ref.	1.07 (0.88, 1.29)	0.494	**1.90 (1.63, 2.22)**	**<0.001**
Model 2	Ref.	1.13 (0.93, 1.36)	0.215	**1.43 (1.21, 1.67)**	**<0.001**
365-day mortality					
Crude	Ref.	0.87 (0.73, 1.03)	0.104	**1.46 (1.27, 1.67)**	**<0.001**
Model 1	Ref.	1.04 (0.87, 1.23)	0.683	**1.75 (1.52, 2.01)**	**<0.001**
Model 2	Ref.	1.08 (0.91, 1.28)	0.381	**1.33 (1.15, 1.54)**	**<0.001**

a Bold for *p* < 0.05.

b Adjusted for age and gender.

c Model 1 + SOFA, APS-III, invasive ventilation, CRRT, and RRT.

### *Post-hoc* exploration in the high-risk sub-phenotype

Given established evidence linking blood gas parameters to ICU mortality (25–28), we investigated blood gas ranges over the initial 12-h period to elucidate mechanisms underlying the elevated risk in Cluster 2. Non-survivors exhibited more blood gas derangements, including lower pH, elevated PCO₂, higher lactate, and reduced base excess, reflecting severe hypoxemia and metabolic imbalance (Supplementary Figure S6).

**Figure 3:**
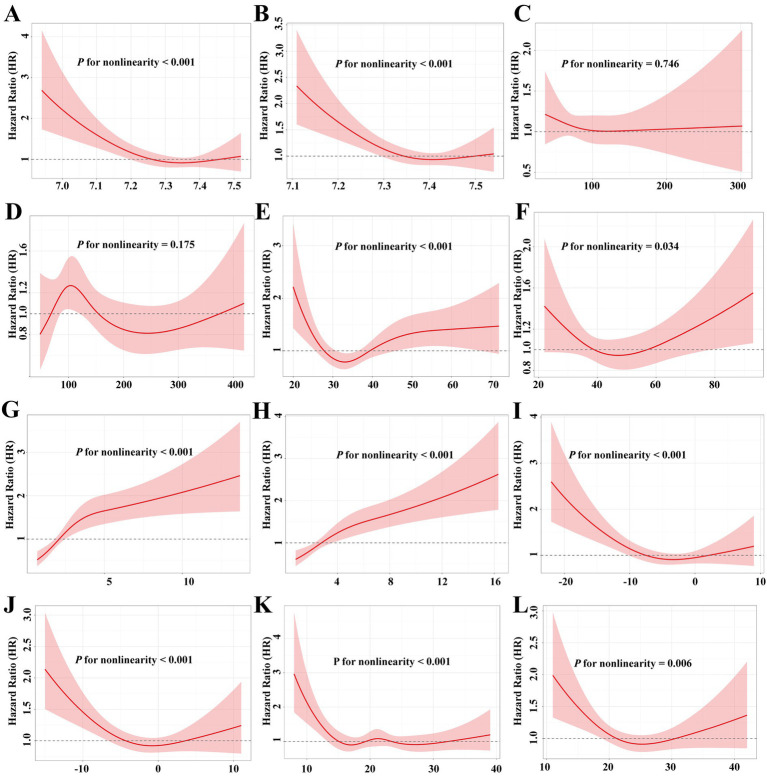
Dose response association of blood gas range with ICU mortality. RCS models of relative ratios of blood gas value and ICU mortality. **(A,B)** pH; **(C,D)** PO_2_; **(E,F)** PCO_2_; **(G,H)** lactate; **(I,J)** base excess; **(K,L)** total PCO_2_. Solid lines, hazard ratios; shadow, 95% confidence interval. Model adjusted for age, sex, SOFA score.

Considering potential complex, non-linear relationships, we employed RCS to model the continuous association between blood gas parameter ranges and ICU mortality. As shown in Figure 3, most blood gas parameters (pH, PCO₂, lactate, base excess, total CO₂) exhibit significant non-linear associations with ICU mortality, suggesting that abnormal blood gas values may correlate with elevated ICU mortality risk. Conversely, the non-linear relationship between PO₂ and ICU mortality risk was not found.

**Figure 4:**
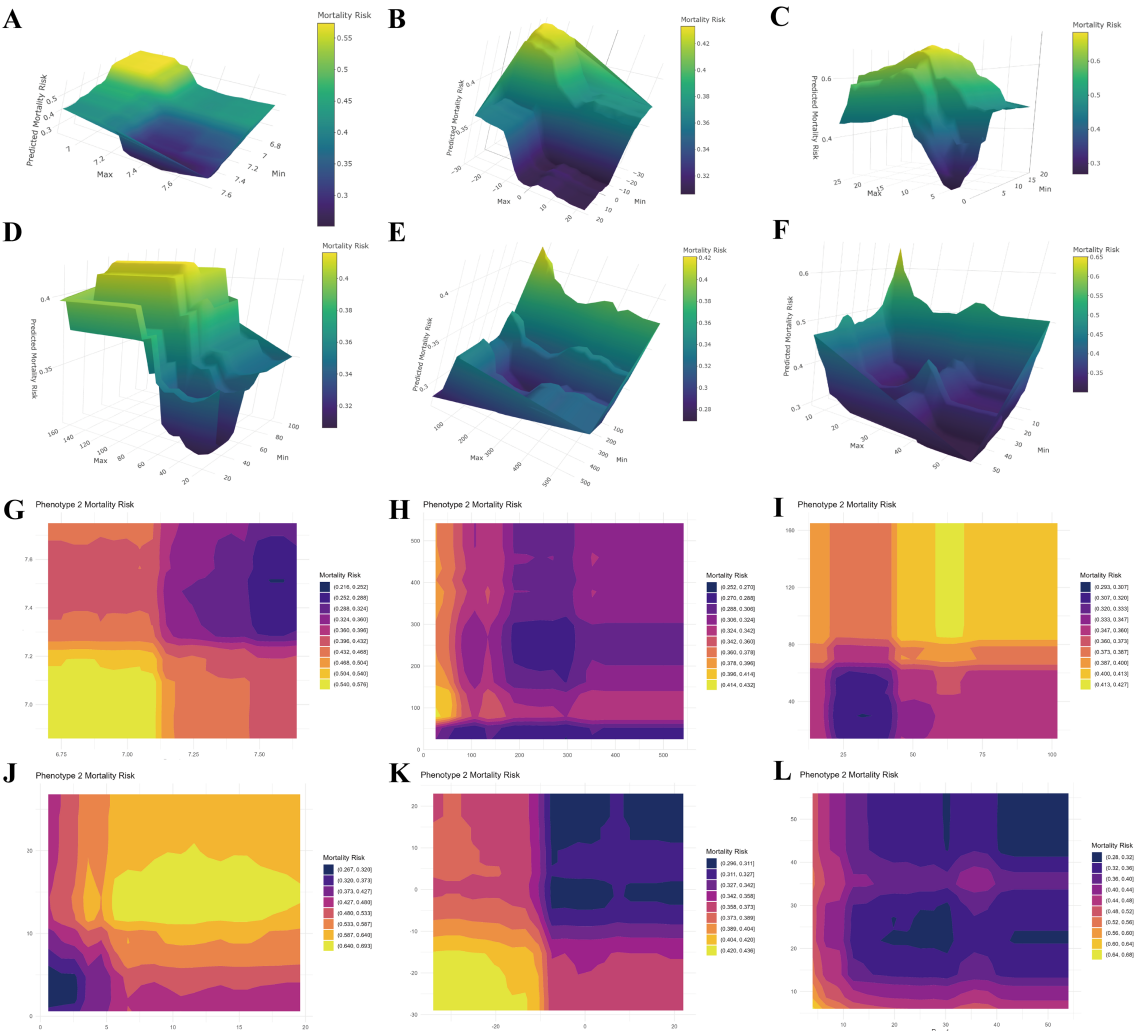
Partial dependence plots for distinct random forest models fitted for the high-risk subgroup. **(A–F)** 3D visualizations between blood gas ranges and ICU mortality risk. **(G–L)** Corresponding 2D heatmaps. **(A,G)** pH; **(B,H)** PO_2_; **(C,I)** PCO_2_; **(D,J)** lactate; **(E,K)** base excess; **(F,L)** total PCO_2_.

Using PDP algorithms, we further visualized the relationship between blood gas ranges and ICU mortality within Cluster 2 (Figure 4). ThreeD and heatmap representations revealed distinct response patterns across blood gas ranges. We also identified the value ranges for each blood gas parameter associated with the lowest range of model-predicted mortality risk, defining a recommended “safe zone” for the high-risk subgroup: pH 7.32–7.64, PO_2_ 25.00–324.32 mmHg, PCO_2_ 21.94–53.74 mmHg, lactate 0.6–7.49 mmol/L, base excess −7.47 to 23.00 mEq/L, total PCO_2_, 43.47–56.00 mEq/L.

## Discussion

In this study, we identified three distinct subgroups of SATP based on vital sign trajectories during the first 12 h following ICU admission. Cluster 1 was characterized by hypertension; Cluster 2 exhibited high heart rate, high respiratory rate, low SpO₂, and hyperinflammatory features; and Cluster 3 displayed a hypo-inflammatory profile. Among these, Cluster 2 was associated with the highest mortality risk, indicating that early vital signs trajectories may reflect divergent underlying pathophysiological states (24, 29). Furthermore, we identified a non-linear relationship between blood gas parameters and ICU mortality in Cluster 2, and used PDP to define the blood gas ranges associated with the lowest mortality risk during the initial 12-h ICU period.

Vital sign trajectory analysis has been increasingly applied to SATP subphenotyping, revealing patterns of disease evolution that can inform individualized treatment strategies (14). Interestingly, similar to our findings, Bhavani et al. (14) identified four clinical trajectory-based subgroups among sepsis patients. Their Group A demonstrated hyperthermia, tachycardia, tachypnea, and hypotension; Group B shared similar features but with hypertension; Groups C and D exhibited lower temperature, heart rate, and respiratory rate, differing in blood pressure profiles. Group A had the highest 30-day mortality, consistent with our high-risk Cluster 2. Our study extends this line of inquiry by focusing specifically on a clinically distinct and high-risk subgroup. This focused approach shifts the emphasis from general sepsis heterogeneity to deep phenotyping within a well-defined pathological entity (17, 30). The differences in subgroup classification may stem from the unique pathophysiology of thrombocytopenic sepsis or the refined temporal resolution of our hourly data, which likely captures more acute, pre-ICU deterioration patterns.

While trajectory modeling effectively stratified risk, we further investigated the role of early blood gas derangements in driving mortality within the high-risk Cluster 2. The critical importance of blood gas analysis in the ICU management of sepsis extends far beyond its routine application in monitoring ventilation and acid–base status (26). For instance, Liang et al. (31) demonstrated that lactate trajectories are significantly associated with mortality risk among early-survival patients with sepsis. Multiple studies have reported non-linear relationships between biochemical markers and sepsis outcomes (28, 32, 33). For instance, Hyun et al. (28) used RCS models to demonstrate that both low and high PO₂ levels on ICU days 2 and 3 were associated with increased 28-day mortality. Similarly, our RCS analyses revealed U- or sigmoid-shaped relationships between blood gas ranges and ICU mortality, reinforcing the concept of a physiological “sweet spot.” These findings highlight that both the direction and magnitude of blood gas abnormalities significantly impact ICU mortality in SATP, underscoring the need for subgroup-specific management (31).

By applying PDP algorithms, we quantified optimal ranges for blood gas parameters that correspond to the lowest predicted mortality risk in high-risk SATP patients. This approach shifts the focus from risk prediction to model-informed physiological insights, thereby providing a data-driven framework for understanding optimal physiological ranges in this high-risk subphenotype. Notably, the PDP-derived ranges were broadly consistent with the RCS models, reinforcing their clinical relevance. The results we observed between blood gas ranges and mortality within the high-risk subgroup suggest a critical principle: in sepsis, it may be more accurate to view normal values as context-dependent rather than universal, with their appropriateness largely shaped by a patient’s underlying Vital signs status.

An inherent challenge in observational critical care research is the presence of confounding by indication. In our study, we addressed potential confounders through multivariable Cox regression adjustment, incorporating a comprehensive range of demographic, severity, comorbidity, and early intervention variables. In this context, the application of more advanced causal inference methods, such as propensity score weighting, can further strengthen the validity of observational findings (34). While our primary analysis did not employ propensity score weighting, the consistency of our findings across multiple levels of covariate adjustment, along with the narrow confidence intervals observed in our Cox models, suggests that the observed mortality differences across trajectory subgroups are unlikely to be entirely explained by measured confounders. Future studies aiming to validate our “trajectory-to-targets” framework could benefit from incorporating propensity score-based methods to further mitigate confounding and strengthen causal inferences regarding the optimal blood gas targets identified for the high-risk phenotype.

Our study has several strengths. To our knowledge, it is the first to derive SATP subgroups using multivariate longitudinal vital sign trajectories from the early ICU period. Moreover, by integrating machine learning with trajectory-based phenotyping, we characterized heterogeneous physiological responses within a high-risk subgroup, providing a novel framework for precision management. Several limitations should be acknowledged. First, due to the inherent limitations of the MIMIC-IV database, we cannot ascertain that all patients met the diagnostic criteria for SATP strictly within the first 12 h of ICU admission. Second, as our model was developed and tested within a single database, external validation in independent cohorts is needed to confirm generalizability. Third, although we adjusted for numerous confounders, residual confounding remains possible. Fourth, the MIMIC-IV database lacks granular treatment details (e.g., ventilator settings, vasopressor use), which directly influence blood gas parameters. This introduces potential confounding by indication, meaning we cannot fully distinguish whether the observed associations reflect the benefits of specific blood gas ranges or the underlying illness severity that prompted treatment. Despite these limitations, our study provides a hypothesis-generating framework for future prospective validation.

Future research should explore joint trajectory modeling of physiological and laboratory parameters to provide a more comprehensive assessment of sepsis pathophysiology (35). In addition, given the evolution of ICU management protocols and patient outcomes during the COVID-19 pandemic, the generalizability of our findings to contemporary, post-pandemic cohorts requires external validation (36).

## Conclusion

In summary, this study unveiled early vital sign trajectory-based subgroups of SATP, identified a high-risk subgroup, and revealed non-linear associations between blood gas parameters and ICU mortality. By defining optimal ranges for key blood gas variables, we provided a data-driven blueprint for precision physiological management in high-risk SATP patients. Future research should focus on integrating these phenotyping approaches into real-time clinical decision support systems. A critical next step will be a randomized controlled trial evaluating whether adherence to model-derived physiological ranges improves patient outcomes compared to standard care.

## Data Availability

The raw data supporting the conclusions of this article will be made available by the authors, without undue reservation.

## Glossary

ICU: Intensive care unit
SATP: Sepsis-associated thrombocytopenia
MIMIC-IV: Medical Information Mart for Intensive Care IV
HR: Heart rate
RR: Respiratory rate
SBP: Systolic blood pressure
DBP: Diastolic blood pressure
MAP: Mean arterial pressure
SpO_2_: Oxygen saturation
WBC: White blood cell count
RBC: Red blood cell count
RDW: Red cell distribution width
BUN: Blood urea nitrogen
PO_2_: Arterial partial oxygen pressure
Total PCO_2_: Total pressure of carbon dioxide
PCO_2_: Partial pressure of carbon dioxide
PT: Prothrombin time
PTT: Partial thromboplastin time
INR: International normalized ratio
SOFA: Sequential organ failure assessment
GBMTM: Group-based multi-trajectory model
HRs: Hazard ratios
RCS: Restricted cubic splines
RF: Random forest
PDP: Partial dependence plot

## References

[1] Singer M Deutschman CS Seymour CW Shankar-Hari M Annane D Bauer M . The third international consensus definitions for sepsis and septic shock (sepsis-3). JAMA. (2016) 315:801–10. doi: 10.1001/jama.2016.0287, 26903338 PMC4968574

[2] Shankar-Hari M Phillips GS Levy ML Seymour CW Liu VX Deutschman CS . Developing a new definition and assessing new clinical criteria for septic shock: for the third international consensus definitions for sepsis and septic shock (sepsis-3). JAMA. (2016) 315:775–87. doi: 10.1001/jama.2016.0289, 26903336 PMC4910392

[3] Seymour CW Liu VX Iwashyna TJ Brunkhorst FM Rea TD Scherag A . Assessment of clinical criteria for sepsis: for the third international consensus definitions for sepsis and septic shock (sepsis-3). JAMA. (2016) 315:762–74. doi: 10.1001/jama.2016.0288, 26903335 PMC5433435

[4] Rhodes A Evans LE Alhazzani W Levy MM Antonelli M Ferrer R . Surviving sepsis campaign: international guidelines for management of sepsis and septic shock: 2016. Intensive Care Med. (2017) 43:304–77. doi: 10.1007/s00134-017-4683-6, 28101605

[5] Angus DC van der Poll T. Severe sepsis and septic shock. N Engl J Med. (2013) 369:840–51. doi: 10.1056/NEJMra1208623 23984731

[6] Claushuis TA van Vught LA Scicluna BP Wiewel MA Klein Klouwenberg PM Hoogendijk AJ . Thrombocytopenia is associated with a dysregulated host response in critically ill sepsis patients. Blood. (2016) 127:3062–72. doi: 10.1182/blood-2015-11-680744, 26956172

[7] Thiery-Antier N Binquet C Vinault S Meziani F Boisramé-Helms J Quenot JP . Is thrombocytopenia an early prognostic marker in septic shock? Crit Care Med. (2016) 44:764–72. doi: 10.1097/ccm.0000000000001520, 26670473

[8] Sharma B Sharma M Majumder M Steier W Sangal A Kalawar M. Thrombocytopenia in septic shock patients—a prospective observational study of incidence, risk factors and correlation with clinical outcome. Anaesth Intensive Care. (2007) 35:874–80. doi: 10.1177/0310057x0703500604, 18084977

[9] Knöbl P. Thrombocytopenia in the intensive care unit: diagnosis, differential diagnosis, and treatment. Med Klin Intensivmed Notfmed. (2016) 111:425–33. doi: 10.1007/s00063-016-0174-8, 27255225 PMC7095953

[10] Levi M Löwenberg EC. Thrombocytopenia in critically ill patients. Semin Thromb Hemost. (2008) 34:417–24. doi: 10.1055/s-0028-1092871 18956281

[11] Jonsson AB Rygård SL Hildebrandt T . Thrombocytopenia in intensive care unit patients: a scoping review. Acta Anaesthesiol Scand. (2021) 65:2–14. doi: 10.1111/aas.13699, 32916017

[12] Raque VX Carlos SJ Eduardo RR Rafael BH Ángeles RML Adriana RC . Modification of immunological features in human platelets during sepsis. Immunol Investig. (2018) 47:196–211. doi: 10.1080/08820139.2017.1413113, 29220594

[13] Romare C Anderberg P Sanmartin Berglund J Skär L. Burden of care related to monitoring patient vital signs during intensive care; a descriptive retrospective database study. Intensive Crit Care Nurs. (2022) 71:103213. doi: 10.1016/j.iccn.2022.103213, 35184970

[14] Bhavani SV Semler M Qian ET Verhoef PA Robichaux C Churpek MM . Development and validation of novel sepsis subphenotypes using trajectories of vital signs. Intensive Care Med. (2022) 48:1582–92. doi: 10.1007/s00134-022-06890-z, 36152041 PMC9510534

[15] Bhavani SV Robichaux C Verhoef PA Churpek MM Coopersmith CM. Using trajectories of bedside vital signs to identify COVID-19 subphenotypes. Chest. (2024) 165:529–39. doi: 10.1016/j.chest.2023.09.020, 37748574 PMC10925543

[16] Reddy K Sinha P O'Kane CM Gordon AC Calfee CS McAuley DF. Subphenotypes in critical care: translation into clinical practice. Lancet Respir Med. (2020) 8:631–43. doi: 10.1016/s2213-2600(20)30124-7, 32526190

[17] Bhavani SV Wolfe KS Hrusch CL Greenberg JA Krishack PA Lin J . Temperature trajectory subphenotypes correlate with immune responses in patients with sepsis. Crit Care Med. (2020) 48:1645–53. doi: 10.1097/ccm.0000000000004610, 32947475 PMC7837282

[18] Han X Zhu S Zhang H Xia T Gu X. Multiplex cerebrospinal fluid proteomics identifies biomarkers predicting neuropsychiatric symptom progression in mild cognitive impairment and Alzheimer's disease. FASEB J. (2026) 40:e71447. doi: 10.1096/fj.202504014R, 41553038

[19] Anthon CT Pène F Perner A Azoulay E Puxty K van de Louw A . Thrombocytopenia and platelet transfusions in ICU patients: an international inception cohort study (PLOT-ICU). Intensive Care Med. (2023) 49:1327–38. doi: 10.1007/s00134-023-07225-2, 37812225 PMC10622358

[20] Johnson AEW Bulgarelli L Shen L Gayles A Shammout A Horng S . MIMIC-IV, a freely accessible electronic health record dataset. Sci Data. (2023) 10:1. doi: 10.1038/s41597-022-01899-x, 36596836 PMC9810617

[21] Ye W Li Y Zhang M Liu S Li P Tang X . Developing and validating a prognostic model to predict ICU mortality in patients with sepsis-associated thrombocytopenia: a retrospective cohort study based on MIMIC-IV. BMJ Open. (2025) 15:e099691. doi: 10.1136/bmjopen-2025-099691, 40789730 PMC12352255

[22] Wang GH Goodin AJ Reise RC Shorr RI Park T Lo-Ciganic WH. Longitudinal patterns of antidepressant and benzodiazepine use associated with injurious falls in older adults with depression: a retrospective cohort study. BMC Med. (2025) 23:487. doi: 10.1186/s12916-025-04325-2, 40830788 PMC12366386

[23] Leisch F. FlexMix: a general framework for finite mixture models and latent class regression in R. J Stat Softw. (2004) 11:1–18. doi: 10.18637/jss.v011.i08

[24] Wang Z Wang W Xu J . Development and validation of dynamic clinical subphenotypes in acute pancreatitis patients using vital sign trajectories in intensive care units: a multinational cohort study. Signal Transduct Target Ther. (2025) 10:180. doi: 10.1038/s41392-025-02261-4, 40467599 PMC12137743

[25] Samanta S Singh RK Baronia AK Mishra P Poddar B Azim A . Early pH change predicts intensive care unit mortality. Indian J Crit Care Med. (2018) 22:697–705. doi: 10.4103/ijccm.IJCCM_129_18, 30405279 PMC6201653

[26] Doğan S Aslan S Börta T Sarıaydın M Sayıner HS. Is there an effect of initial and 24-hour blood gas lactate and methemoglobin levels on predicting mortality of patients in the intensive care unit? Life. (2025) 15:373. doi: 10.3390/life15030373, 40141718 PMC11943582

[27] Menteş O Çelik D Yildiz M Kahraman A Cirik MÖ Eraslan Doğanay G . Electrolyte imbalance and its prognostic impact on all-cause mortality in icu patients with respiratory failure. Medicina. (2025) 61:642. doi: 10.3390/medicina61040642, 40282932 PMC12028514

[28] Hyun DG Ahn JH Huh JW Hong SB Koh Y Oh DK . The association of arterial partial oxygen pressure with mortality in critically ill sepsis patients: a nationwide observational cohort study. Crit Care. (2024) 28:187. doi: 10.1186/s13054-024-04960-w, 38816883 PMC11140987

[29] Wang HC Fang CC Huang CH Gao JW Chen JH Tsai CL. Factors associated with overall and high-risk return visits to the emergency department: a vital sign trajectory approach. BMC Emerg Med. (2025) 25:57. doi: 10.1186/s12873-025-01211-1, 40221661 PMC11993975

[30] Bhavani SV Spicer A Sinha P Malik A Lopez-Espina C Schmalz L . Distinct immune profiles and clinical outcomes in sepsis subphenotypes based on temperature trajectories. Intensive Care Med. (2024) 50:2094–104. doi: 10.1007/s00134-024-07669-0, 39382693 PMC12674214

[31] Liang Z Zhao M Liu K Liang W Luo S Guan J . Lactate trajectories in early survivors of sepsis: a new lens on mortality risk. Shock. (2025) 64:386–96. doi: 10.1097/shk.0000000000002653, 40720292 PMC12435258

[32] Li L Li L Zhao Q Liu X Liu Y Guo K . High serum magnesium level is associated with increased mortality in patients with sepsis: an international, multicenter retrospective study. MedComm. (2024) 5:e713. doi: 10.1002/mco2.713, 39290253 PMC11406045

[33] Ge Y Wang Z Ma Y Zhang C. Prognostic value of the glucose-to-albumin ratio in sepsis-related mortality: a retrospective ICU study. Diabetes Res Clin Pract. (2025) 224:112217. doi: 10.1016/j.diabres.2025.112217, 40345593

[34] Zhu S Zheng Z Wang L Luo G Zhang Y Jia T . Association between loop diuretics and mortality in patients with cardiac surgery-associated acute kidney injury: a retrospective propensity score-weighted analysis. Anesth Analg. (2024) 139:124–34. doi: 10.1213/ane.0000000000006748, 38009938

[35] Zhu S Lu P Liu Z Li S Li P Wei B . Longitudinal hemoglobin trajectories and acute kidney injury in patients undergoing cardiac surgery: a retrospective cohort study. Front Cardiovasc Med. (2023) 10:1181617. doi: 10.3389/fcvm.2023.1181617, 37265564 PMC10229827

[36] Wang L Zheng Z Zhu S Luo G Gao B Ma Y . Changes in early postoperative outcomes and complications observed in a single center during the 2022 COVID-19 pandemic wave in China: a single-center ambispective cohort study. Chin Med J. (2023) 136:1708–18. doi: 10.1097/cm9.0000000000002724, 37310058 PMC10344551

