# Supplementary material for: From vital sign trajectories to data-driven targets: defining exploratory blood gas ranges in sepsis-associated thrombocytopenia
Source: Front Med (Lausanne). 2026 Apr 9;13:1801744. doi: 10.3389/fmed.2026.1801744 (PMC13102763; doi:10.3389/fmed.2026.1801744)
Supplement: Supplementary file 1 [file Supplementary_file_1.pdf]

From Vital Sign Trajectories to Data-Driven Targets: Defining Exploratory Blood Gas Ranges in Sepsis-Associated Thrombocytopenia

Shihao Jin[1], Haotian Hu[1], Xue Wang[1], Xuan Wei[1], Changjie Wang[1], Peisen Ding[2], Pengfei Fan[1], Sinan Gao[1], Xiaojing Dou[1], Bing Wang[1 *]

1 Surgical Intensive Care Unit, Tianjin First Central Hospital, Tianjin, China.

2 Department of Science, Technology and Education, Tianjin First Central Hospital, Tianjin, China.

* Corresponding Author – Bing Wang, PhD

Tel.: +86 022 23629669

E-mail address: egenbing@163.com;

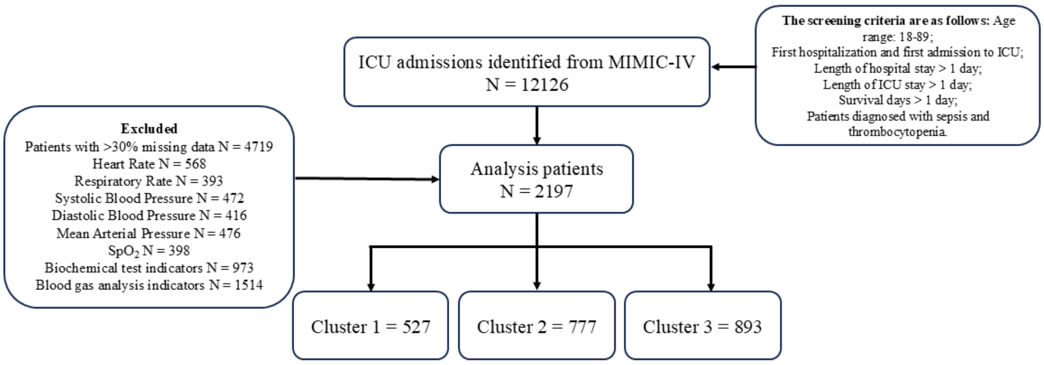

Fig. S1 Schematic diagram of study population selection steps

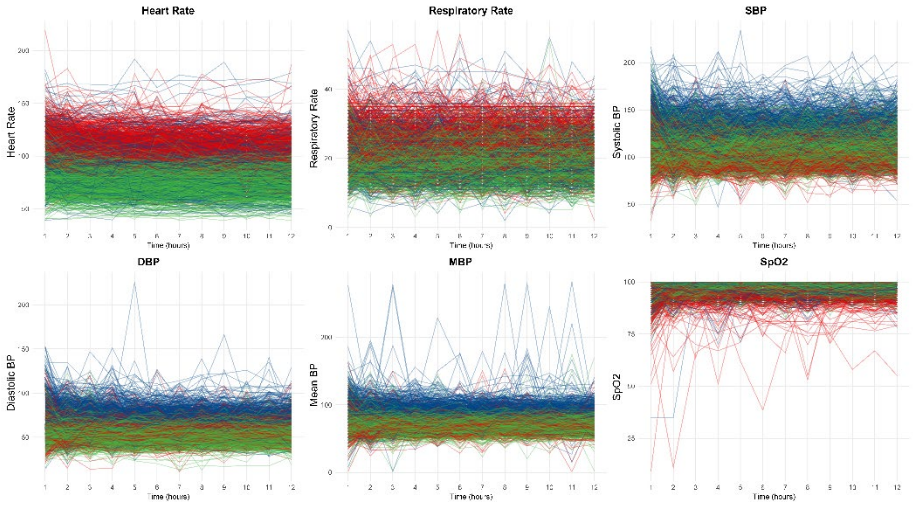

Fig. S2 Individual trajectories of physiological parameters over 12 hours

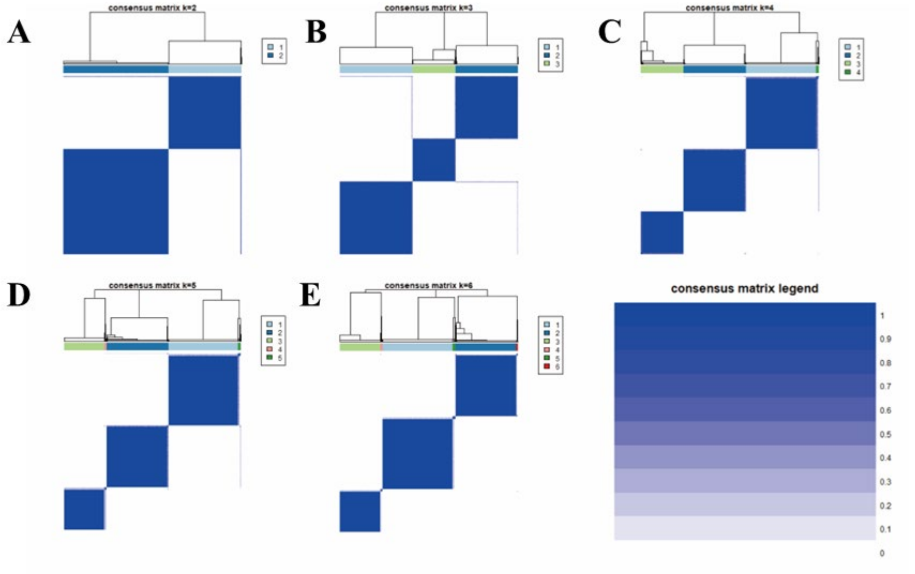

Fig. S3 Consensus matrices (K= 2-6). The consensus matrices for SATP subgroups (K=2-6) were presented in the form of microarrays (Supplementary Fig. 3A-E). These matrices capture consensus values ranging from 0 (indicating instances where subgroups were never clustered together) to 1 (indicating instances where subgroups were always clustered together). The visualization of these matrices was represented as heatmaps, with colors ranging from white (value=0) to dark blue (value=1). The arrangement of the consensus matrices followed the order of consensus clustering, which is visually depicted as a dendrogram positioned above the heatmap. Notably, when K=2-6, distinct groups within the heatmap exhibited well-defined boundaries, which signifies the robustness and stability of the identified subgroups. These clear boundaries underscore the consistency in the clustering results, where subgroups within the same group consistently clustered together across multiple iterations, as indicated by the high consensus values in the corresponding cells of the heatmap.

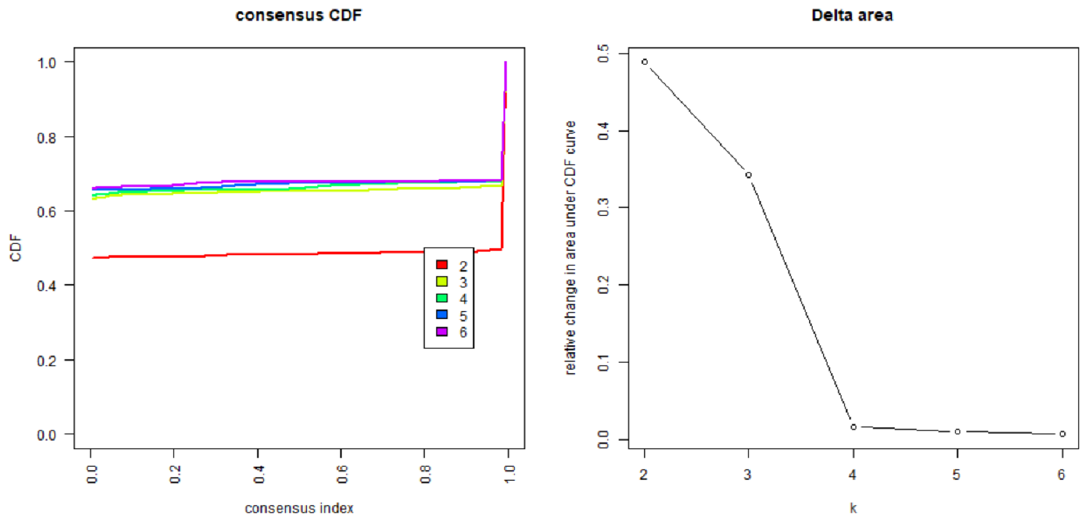

Fig. S4 Consensus cumulative distribution function (CDF) plot and delta area plot. The CDF plot, as shown in Supplementary Fig. 4a, illustrates the consensus distributions across different values of K, ranging from K=2 to 6. When comparing these curves, it becomes evident that K=2-6 exhibit desirable shape, characterized by the steep ascent of values in the range between 0 and 1. This steep climb in the CDF curve signifies a strong consensus among the subgroups within these clusters. The delta area curve, represented in Supplementary Fig. 4b, quantifies the relative change in area beneath the CDF curve when moving from K to K-1. Notably, the delta area curve highlights K=3 as a distinct elbow point. This finding indicates that K=3 provides a meaningful level of cluster stability and structure, making it a valuable choice for identifying subgroups within the dataset.

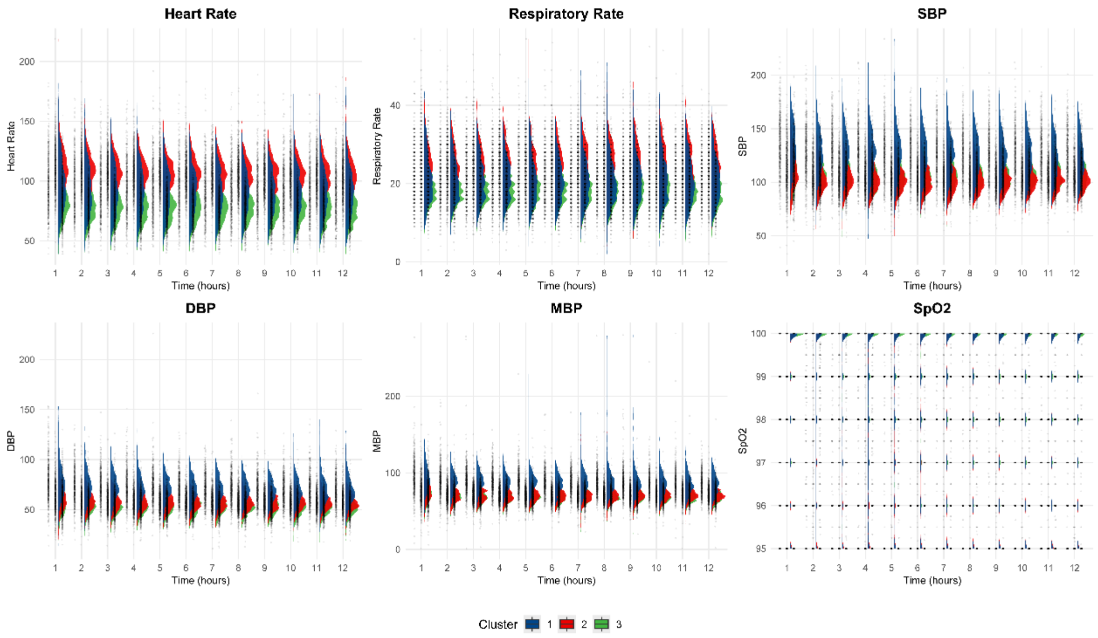

Fig. S5 Distribution of vital signs of STAP patents in the first 12 hours after ICU admission. From left to right is the distribution of HR, RR, SBP, DBP, SpO_2 based on the phenotypes at 0-12 hours.

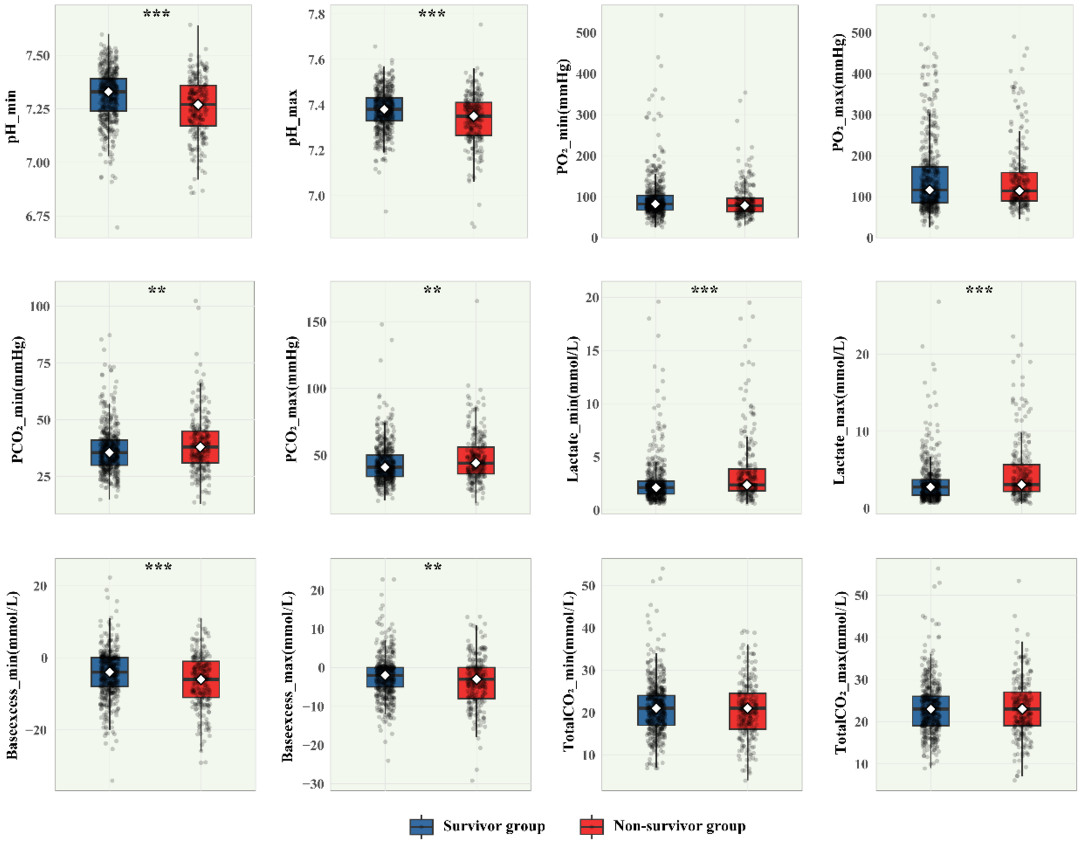

Fig. S6 Comparison of blood gas analysis extremes within the ICU survival/non-survival subgroups during the first 12 hours after ICU admission in cluster 2. *$P < 0.05$; **$P<0.01$; ***$P < 0.001$

Table S1 Comparison of clinical indicators across different subgroups

Variables	Cluster 1	Cluster 2	Cluster 3	*P*
Age	61.63 ± 14.43	61.55 ± 15.42	67.39 ± 14.12	< 0.001
Hospital length of stay (Day)	20.59 ± 18.05	19.57 ± 18.82	17.66 ± 15.24	0.005
ICU length of stay (Day)	9.01 ± 10.22	9.63 ± 10.53	7.60 ± 8.37	< 0.001
WBC	13.58 ± 10.41	15.27 ± 12.08	13.75 ± 8.89	0.003
Hematocrit	33.33 ± 7.77	32.57 ± 7.19	31.25 ± 6.69	< 0.001
MCH	30.31 ± 3.00	30.17 ± 3.19	30.52 ± 2.84	0.056
MCV	92.14 ± 7.72	93.22 ± 8.69	93.64 ± 7.90	0.003
MCHC	32.92 ± 1.79	32.39 ± 1.76	32.63 ± 1.59	< 0.001
RDW	15.49 ± 2.23	16.05 ± 2.73	15.80 ± 2.67	< 0.001
Platelet	191.67 ± 128.33	192.35 ± 130.60	183.08 ± 110.75	0.238
RBC	3.64 ± 0.89	3.53 ± 0.83	3.37 ± 0.78	< 0.001
Creatinine	1.81 ± 2.20	1.94 ± 1.64	1.88 ± 1.78	0.457
BUN	31.65 ± 26.37	36.97 ± 26.92	36.72 ± 27.82	< 0.001
Anion gap	15.82 ± 4.90	16.77 ± 5.87	15.21 ± 5.03	< 0.001
Total calcium	8.24 ± 1.00	7.90 ± 1.04	8.11 ± 0.90	< 0.001
PT	17.21 ± 8.95	19.69 ± 12.67	18.39 ± 10.02	< 0.001
PTT	38.92 ± 22.80	41.12 ± 21.94	42.80 ± 27.04	0.015
INR	1.58 ± 0.84	1.80 ± 1.15	1.70 ± 0.97	< 0.001
Chloride	103.35 ± 7.02	102.86 ± 7.36	103.76 ± 7.40	0.043

Glucose	160.84 ± 79.83	159.53 ± 104.77	155.19 ± 76.85	0.432
Potassium	4.24 ± 0.91	4.27 ± 0.83	4.22 ± 0.81	0.452
Sodium	137.99 ± 5.89	137.20 ± 6.17	137.59 ± 5.81	0.061
Bicarbonate	21.89 ± 4.86	20.44 ± 5.81	21.58 ± 5.19	< 0.001
Hemoglobin	10.97 ± 2.53	10.55 ± 2.36	10.34 ± 2.26	< 0.001
Magnesium	2.02 ± 0.73	1.99 ± 0.55	1.99 ± 0.45	0.47
Phosphate	4.00 ± 1.69	4.20 ± 1.92	4.15 ± 1.70	0.123
SOFA	8.87 ± 4.42	10.91 ± 4.65	9.38 ± 4.07	< 0.001
pH	7.37 ± 0.10	7.32 ± 0.12	7.35 ± 0.10	< 0.001
PO_2	140.05 ± 92.18	120.15 ± 79.68	150.31 ± 98.41	< 0.001
PCO_2	40.50 ± 12.51	41.83 ± 14.91	41.42 ± 12.47	0.21
Base excess	-2.04 ± 5.20	-4.05 ± 6.54	-2.59 ± 5.46	< 0.001
Lactate	2.50 ± 2.06	3.34 ± 2.90	2.37 ± 1.78	< 0.001
Total CO_2	23.60 ± 5.52	22.23 ± 6.57	23.35 ± 5.89	< 0.001

Table S2 Fit statistics of GBMTM from 2 to 6 subgroups

Model	No. of Class	Loglik	AIC	BIC	ICL	Entropy	% Class 1	% Class 2	% Class 3	% Class 4	% Class 5	% Class 6
GBMTM-2	2	-210782.27	421662.53	422063.34	423867.36	0.9506	62.81	37.19	/	/	/	/
GBMTM-3	3	-203243.27	406634.54	407239.84	409821.19	0.9554	23.99	35.36	40.65	/	/	/
GBMTM-4	4	-198888.74	397975.48	398785.28	401372.17	0.9646	23.71	24.85	31.45	19.99	/	/
GBMTM-5	5	-195974.35	392196.71	393211.00	396270.53	0.9639	21.85	21.48	12.79	22.53	21.35	/
GBMTM-6	6	-193026.16	386350.32	387569.10	391045.68	0.9632	19.34	12.74	18.16	15.34	17.93	16.49
